# Growth arrest of *Mycobacterium tuberculosis* in acidic environments enhances their survival of antibiotic treatment

**DOI:** 10.1371/journal.pbio.3003857

**Published:** 2026-06-22

**Authors:** Eun Seon Chung, William C. Johnson, Maliwan Kamkaew, Timothy A. Fitzgerald, Morgan E. McNellis, Trever C. Smith, Srinivasan Vijay, Nguyen Thuy Thuong Thuong, Shumin Tan, Bree B. Aldridge

**Affiliations:** 1 Department of Molecular Biology and Microbiology, Tufts University School of Medicine, Boston, Massachusetts, United States of America; 2 Tufts University Graduate School of Biomedical Sciences, Boston, Massachusetts, United States of America; 3 Department of Microbial Pathogenesis and Immunology, Texas A & M University School of Medicine, Bryan, Texas, United States of America; 4 Oxford University Clinical Research Unit, Ho Chi Minh City, Vietnam; 5 Centre for Tropical Medicine and Global Health, Nuffield Department of Medicine, University of Oxford, Oxford, United Kingdom; 6 Stuart B. Levy Center for Integrated Management of Antimicrobial Resistance, Tufts University, Boston, Massachusetts, United States of America; 7 Department of Biomedical Engineering, Tufts University School of Engineering, Medford, Massachusetts, United States of America; National Centre for Biological Sciences, INDIA

## Abstract

The ability of *Mycobacterium tuberculosis* (Mtb) to dynamically adjust its growth behavior in response to host environments is critical for survival under immune and drug stress, but how these behaviors shift at the single-cell level remains poorly understood. Here, using high-resolution single-cell analysis, we show that Mtb adapts to acidic conditions by increasing the proportion of bacteria in a growth-arrested state, rather than uniformly slowing the growth rate of the entire population. This nongrowing subpopulation exhibits enhanced tolerance to ethambutol, highlighting its role in drug survival. Clinical strains displayed higher proportions of growth-arrested cells under both neutral and acidic conditions, suggesting that growth arrest may serve as one of the strategies for persistence during infection. While the PhoPR two-component system partially regulates this state, our RNA sequencing analysis revealed additional transcriptional regulators that are upregulated following acidic adaptation and may contribute to entry into the growth-arrested state and increased tolerance to ethambutol. Our study demonstrates that increasing the proportion of nongrowing subpopulations is an active adaptive strategy that can influence antibiotic susceptibility under acidic conditions, offering new perspectives for targeting bacterial heterogeneity in tuberculosis therapy.

## Introduction

Tuberculosis (TB), caused by infection with the bacterium *Mycobacterium tuberculosis* (Mtb), requires a lengthy multi-drug regimen to cure [1]. One of the key challenges in TB treatment is the complex biology of Mtb bacilli, with heterogeneity in Mtb cell state thought to be a major driver of treatment failure [2–4]. During infection, Mtb encounters diverse conditions and environmental cues, including acidic pH in the phagosome, lipid-rich environments, and exposure to reactive oxygen species and other immune defenses [5–8]. As resident bacteria adapt to this range of conditions, some cells’ growth and metabolic states change, generating phenotypically distinct subpopulations that may survive, evading immune clearance and drug killing [5,9–13]. Understanding how drug-tolerant subpopulations of Mtb form in host microenvironments is crucial for improving TB treatment strategies.

One of the earliest stressors Mtb encounters during infection is the acidic pH of the macrophage phagosome [14]. Before immune activation in macrophages, Mtb inhibits phagolysosome formation and resides in a mildly acidic environment (pH ~ 6.2) [15–17]. Mtb adapts to this mildly acidic stress through gene regulation and metabolic shifts that lead to bulk-level growth slowing or growth arrest, which allows Mtb to persist within a host environment [18–22]. Although these population-level studies provide insights into Mtb’s metabolic plasticity, most studies examine growth rate or growth arrest through bulk measurements, such as optical density (OD_600_) and colony-forming unit (CFU) assays. We therefore lack an understanding of Mtb growth behaviors and the changes of subsets of cells during adaptation to host-relevant growth conditions.

Recent advances in single-cell imaging techniques have revealed substantial heterogeneity in mycobacterial growth dynamics, with individual cells exhibiting variations in growth rates, cell cycle timing, and cell size, which can affect drug susceptibility [23–30]. We examined whether growth slowing at acidic pH is uniform across the population or whether there is cell-to-cell variation in growth response. We hypothesized that variability in subpopulation responses may be a source of heterogeneity that contributes to such growth slowing and pH adaptation, leading to persistence. Our study revealed that, rather than uniformly slowing growth, Mtb exhibits a bimodal response in which a subset of cells continues to grow at the same rate while another subset converts to a nongrowing state. Clinical strains were particularly prone to enter a growth arrested state. We find that nongrowing states are present even in rich, neutral growth conditions, suggesting that Mtb growth arrest is a typical bacterial state and is not due to resource limitations. We challenged Mtb with antibiotic stress and observed that nongrowing cells exhibit increased tolerance to the cell wall-targeting antibiotic ethambutol. We observed several regulators that modulate this growth behavior under acidic pH conditions, including the two-component system PhoPR, which we also demonstrated contributes to growth-arrest-mediated ethambutol tolerance. Our findings suggest that Mtb employs phenotypic diversification by modulating the proportion of bacteria in a growth-arrested state, which could influence susceptibility to antibiotic treatment.

## Results

### Growth arrest is the dominant Mtb cell growth-state during adaptation to acidic conditions

Many mycobacterial species, including *M. avium*, *M. chelonae*, *M. marinum*, *M. scrofulaceum*, and *M. fortuitum*, grow without restriction under mildly acidic conditions (~pH 6.0), and some even grow better at an acidic compared to a neutral pH [31]. In contrast, Mtb slows its bulk growth rate in response to an acidic environment [19,32]. A simple explanation for growth slowing is that individual cells reduce their growth rates uniformly.

**Fig 1:**
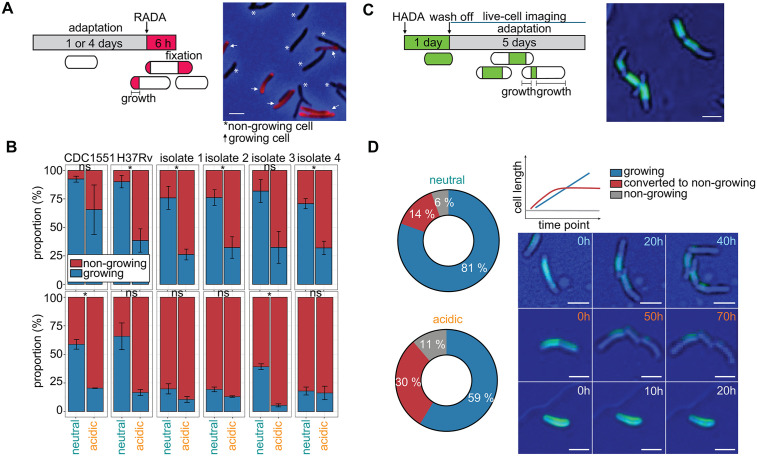
Acidic conditions increase the proportion of nongrowing cells. **(A)** Fixed-cell imaging design using fluorescent D-amino acid (FDAA) for growth measurement. RADA was added for 6 h after 1 or 4 days of adaptation to pH 7.0 or 5.9. Growth is indicated by RADA-labeled length. Scale bar, 2 μm. Asterisks and arrows mark nongrowing cells and growing cells, respectively. **(B)** Proportions of RADA-positive (growing) and -negative (nongrowing) cells in two lab-adapted strains (CDC1551, H37Rv) and four recent clinical isolate strains after 1 (top) or 4 (bottom) days of pH adaptation. Significance was determined by unpaired Student’s *t* tests with Benjamini-Hochberg correction: ns *p* ≥ 0.05, *p < 0.05, **p < 0.01, ***p < 0.001, ****p < 0.0001. Data are from biological triplicates. The data underlying this Figure can be found in https://doi.org/10.5281/zenodo.20438984. **(C)** Live-cell imaging design using HADA labeling (green). CDC1551 cells were pre-labeled for 1 day, washed, and then imaged for 5 days under neutral or acidic conditions. New polar growth appears as unlabeled extensions. Scale bar, 2 μm. **(D)** Classification of subpopulation growth dynamics during pH adaptation. A schematic diagram categorizes cells into three groups: (1) growing and dividing throughout the movie (green), (2) switched from growing to nongrowing (red), or (3) consistently nongrowing (gray) throughout pH adaptation (top right). Representative snapshots of each group from live-cell imaging are shown below. A pie chart shows the distribution under neutral (*n* = 308) and acidic (*n* = 230) conditions from one experiment. Scale bar, 2 μm.

Observations of heterogeneity in mycobacterial growth and metabolic states in vitro and in vivo led us to speculate that Mtb cells may not uniformly slow their growth [23,24,30,33–37]. To measure growth changes in single cells as Mtb adapts to acidic pH conditions, we adapted two lab-adapted Mtb strains (CDC1551 and H37Rv) and four recently isolated strains of Mtb to either pH 5.9 or pH 7.0 conditions for 1–4 days (Materials and methods). To identify actively growing cells after the different adaptation times, we labeled nascent peptidoglycan sites within the Mtb cell wall with the fluorescent D-amino acid (FDAA) RADA during the last 6 h of the adaptation period (Fig 1A). We confirmed that FDAA specifically labels growing cells by using heat-killed cells as a negative control (Fig A in S1 Appendix). The cells were labeled for 6 h, approximately one-third of the average doubling time, to avoid overcounting growing cells by including those that divide during FDAA treatment. Because mycobacteria elongate from the poles, we can analyze the polar staining patterns of the FDAA to measure growth from each pole over the 6-h staining period. Consistent with prior studies demonstrating growth heterogeneity in Mtb [23,26,30], we observed variation in the length of the stained polar regions. Specifically, we observed that a large proportion of cells lacked detectable FDAA incorporation, suggesting these cells were entirely nongrowing (Fig 1A and 1B). After 4 days of acidic adaptation, the proportion of the nongrowing subpopulation among all six strains was 80%–95%, where the proportion was 34%–82% in neutral conditions (Fig 1B). The nongrowing cells remained viable, with 91% of cells resuming growth after acidic adaptation in unbuffered media (Fig B in S1 Appendix and S1 Movie). FDAA labeling did not affect Mtb viability, as the percentage of regrowing cells was similar between HADA-positive (89%) and -negative (91%) groups (Fig B in S1 Appendix). By comparing the proportion of nongrowing cells across different starting ODs, we confirmed that the pH effect on growth arrest is independent of growth phase (Fig C in S1 Appendix). These data suggest that even in “fast-growing” conditions, there is a nonnegligible subpopulation of nongrowing Mtb cells. The proportion of nongrowing cells is higher at pH 5.9 than at pH 7.0 for both days of adaptation for all strains. For example, 59% ± 4.2% of CDC1551 Mtb cells in the neutral condition were growing at day 4, compared with 20% ± 0.2% in the acidic condition (*T* test and Benjamini-Hochberg correction *p*-value = 0.047). Additionally, we observed that the proportion of nongrowing cells increased in a pH-dependent manner, as shown by comparisons between pH 7.0, 6.2, and 5.9 (Fig D in S1 Appendix). Differences in the proportion of nongrowing cells across pH conditions were more pronounced on day 1 than on day 4, which likely reflects differences in bacterial growth phase across the pH conditions, with the clinical isolates reaching the stationary phase earlier than the lab-adapted strains. We consistently observed a higher proportion of nongrowing cells at both pH levels in the clinical isolates when compared to the two lab-adapted strains by day 4 (Fig 1B). This may suggest that having a higher propensity for growth arrest is beneficial for Mtb during infection.

Next, we complemented our fixed-cell imaging assay with a time-lapse pulse-chase imaging experiment to observe the dynamics of the growth adaptation over 5 days (Fig 1C and S2 and S3 Movies). For the fixed-cell experiments, cells were grown for 4 days to analyze nongrowing cells, with cultures beginning to enter the stationary phase by day 4. In contrast, Mtb bacilli in the microfluidic devices experienced continuous media flow and high air permeability, which may slow progression through the growth phases. These differences make it difficult to directly compare the fixed- and live-cell imaging in real time. However, growth arrest in fixed-cell experiments roughly matches the onset of crowding-induced growth limitation in live-cell imaging, which occurs around day 5, when single-cell annotation and tracking become impossible.

We pre-labeled Mtb (CDC1551 strain) with a FDAA (HADA) for 1 day prior to loading the cells into a microfluidic imaging device with neutral (S2 Movie) or acidic (S3 Movie) growth media. We used HADA for the time-lapse imaging because a higher concentration (100 µM) of FDAA was needed to ensure that the whole cell body of every single-cell was labeled without disturbing Mtb growth, and we found that such a high concentration of RADA disrupted cell elongation. We directly observed growth dynamics by measuring the extension of the cell poles as they elongated with new (HADA-negative region) growth occurring after the movie started (Fig 1C, right). We categorized cells tracked over time into three groups based on growth trajectories: “growing,” “nongrowing,” and “converted to nongrowing” (Fig 1D upper right). In both neutral and acidic environments, we confirmed that a proportion of cells slowed growth and entered a nongrowing state (Fig 1D, left). Among cells adapted to pH 7.0 (*n* = 308), 6% were entirely nongrowing (*n* = 17), whereas 14% converted to nongrowing (*n* = 43) during adaptation. These proportions were higher among the cells adapted to pH 5.9 (*n* = 230), 11% (*n* = 26), and 30% (*n* = 69), respectively (Pearson’s Chi-squared test *p*-value = 2.27 × 10^−7^) (Fig 1D, left). Though these proportions do not precisely match the quantities observed by fixed-cell imaging, likely due to differences in assay conditions and methods for growth quantification (see Discussion), they recapitulate the trends in Fig 1B. The time-lapse and fixed-cell imaging data demonstrate that even in growth-permissive conditions, a fraction of the nongrowing population exists, and the proportion of this nongrowing subpopulation is higher in an acidic environment.

### The bulk growth slowdown during acidic pH adaptation is caused by an increase in the proportion of a growth-arrested cell subpopulation

**Fig 2:**
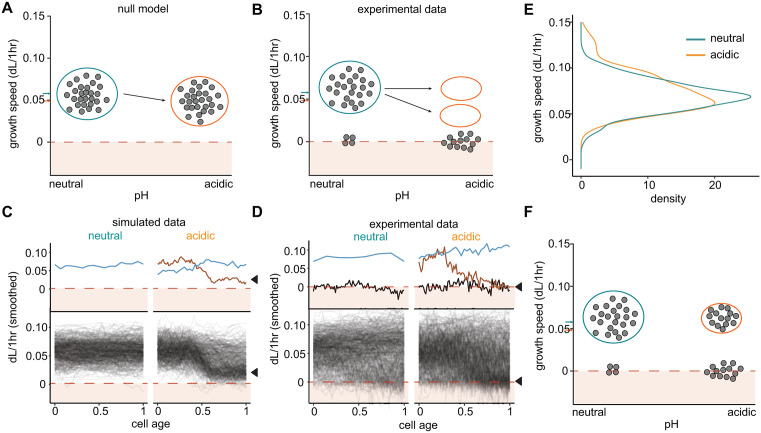
Bulk growth slowdown during acidic adaptation results from an increased proportion of growth-arrested cells. **(A)** Schematic of null model of single-cell growth rate tuning under neutral and acidic conditions. All cells uniformly slow their growth under acidic conditions. Gray dots represent a single-cell. Green and orange circles indicate populations at pH 7.0 and 5.9. Average single-cell growth speeds at pH 7.0 and pH 5.9 are marked on the y-axis. A red dashed line at y = 0 marks growth arrest. (**B)** Schematic of experimentally observed subpopulations of growth-arrested and actively growing cells existing at both pH levels. Growing cells may either maintain (top arrow) or reduce (bottom arrow) their growth rates. **(C–D)** Simulated (C) and experimental (D) data of single-cell growth speeds (y-axis) over normalized cell age from birth to division. Each line represents a smoothed trajectory of an individual cell using a 20-h rolling mean, with colored lines highlighting example cell trajectories. **(E)** Density plot comparing cell growth rates (dL/h) of growing cells between neutral and acidic conditions. The Wilcoxon-rank sum *p*-value = 0.1; *n* = 248 for pH 7.0, *n* = 135 for pH 5.9. **(F)** Revised model: the average growth rate decreases under acidic conditions due to an increased proportion of growth-arrested cells, not slower growth in growing cells. The data underlying this Figure can be found in https://doi.org/10.5281/zenodo.20438984.

Consistent with previous studies, we observed that Mtb growth slows at the population level during adaptation to an acidic environment (Fig EA in S1 Appendix). These data suggest a null model in which the growth of single cells uniformly slows during acidic adaptation, leading to an overall lower average speed compared with growth under neutral conditions (Fig 2A). However, we observed a growth-arrested cell subpopulation in both neutral and acidic conditions (Fig 1B and 1D), suggesting an alternative model in which the bulk growth slowdown is caused by an increase in the proportion of cells in the growth-arrested state (Fig 2B).

To determine which model explains how Mtb adapts to acid stress, we analyzed single-cell growth rates from our time-lapse imaging experiments (Fig 1C). We compared these experimental data to time-lapse imaging single-cell data that were simulated under the null model assumptions. According to the null model, we would expect that Mtb in the neutral condition grows at a fairly constant rate above 0 dL/h throughout the 5 days of adaptation (Fig 2C, left). Instead, in our experimental data, we observed that even in the neutral condition, some cells maintained a growth rate of ~0 dL/h throughout cell tracking (Fig 2D, left), confirming the presence of a subpopulation of nongrowing cells and corroborating our conclusions from Fig 1. Cells adapting to the acidic condition did not slow their growth rate to above 0 dL/h as in the null model (Fig 2C, right, arrow); instead, many entered a completely nongrowing state at ~0 dL/h (Fig 2D, right, arrow).

Additionally, Mtb adapting to acidic pH should slow their growth over the course of tracking time (x-axis), but the final rates should be above 0 dL/h (Fig 2C, right, arrow). In our experimental data on Mtb adapting to acidic conditions, we observed a subpopulation of cells that continued to grow at a rate similar to cells in the neutral condition, regardless of age (Fig 2D, right). We confirmed this quantitatively, finding no significant difference in growth speed between cells that continued to grow in neutral and acidic conditions (Fig 2E, right *p* = 0.12). These data suggest that Mtb adapts to acidic conditions in a bimodal manner (Fig EB in S1 Appendix), with one subpopulation maintaining the same “fast” growth speed while the proportion of growth-arrested cells increases within the population (Fig 2F).

### Nongrowing cells that are induced during acidic adaptation recover better from cell wall-targeting drug treatment

**Fig 3:**
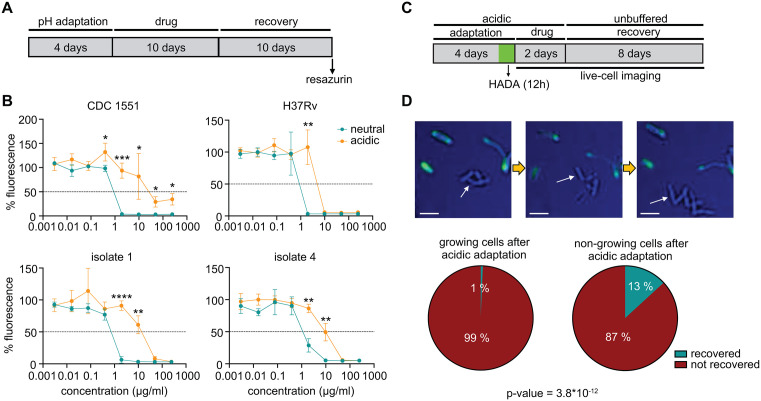
Nongrowing cells arising after acidic adaptation recover better from ethambutol than neutral-adapted cells. **(A)** Experimental design for the resazurin assay. Cells adapted to pH 7.0 or 5.9 for 4 days, treated with ethambutol for 10 days, then recovered drug-free for 10 days. Viability was assessed via 1-h resazurin incubation and fluorescence measurement. **(B)** Four strains (2 lab-adapted, 2 clinical) were tested with varying ethambutol concentrations. Green = neutral-adapted; orange = acidic-adapted. Fluorescence was normalized to untreated controls. Fluorescence level of resazurin reflects metabolic activity of bacterial cells. Data are from biological triplicates. Mean ± SD shown. *P* values were calculated using the two-tailed unpaired *t* test. The data underlying this Figure can be found in S1 Data. **(C)** Experimental design for drug treatment time-lapse imaging. CDC1551 WT was adapted to pH 5.9 for 4 days. HADA was added for the last 12 h. Cells were imaged for 10 days, with 245 µg/ml ethambutol for the first 2 days, followed by fresh media. **(D)** Growth resumption was assessed in HADA-positive (growing, *n* = 736) and HADA-negative (nongrowing, *n* = 152) cells. Fisher’s exact test was used. Arrows highlight previously nongrowing cells resuming growth.

To understand whether the growth-arrested subpopulation was differentially susceptible to drug treatment, we measured the drug responses of Mtb adapted to neutral and acidic conditions across a range of antibiotic types (Fig 3A). Mtb was adapted to either neutral or acidic conditions for four days, then subjected to antibiotic treatment for 10 days. After drug removal by transferring the cell cultures onto agar supplemented with 0.4% charcoal (to inactivate antibiotics), the cells were allowed to recover for an additional 10 days. Following recovery, bacterial survival via metabolic activity was assessed by measuring the fluorescence of resazurin. We measured dose responses for four drugs with different mechanisms of action: ethambutol and isoniazid (cell wall-acting drugs), rifampin (a transcription inhibitor), and linezolid (a translation inhibitor) (Fig 3B, F and G in S1 Appendix).

When Mtb were adapted to acidic conditions, all strains tested exhibited significantly lower susceptibility to ethambutol compared to when they were adapted to neutral conditions (Fig 3B). At 2 µg/ml ethambutol, all four strains in neutral conditions exhibited a drastic reduction in survival, with no recovery observed at 10 µg/ml. Significant differences were detected between neutral and acidic adaptation at both concentrations: at 2 µg/ml, *p*-values were 0.0006, 0.003, 8.8 × 10^−5^, and 0.001 for each strain, respectively; at 10 µg/ml, *p*-values were 0.05, 0.002, and 0.005, excluding the H37Rv strain, respectively (Fig 3B). In contrast, acidic-adapted cells demonstrated robust recovery at 2 µg/ml ethambutol and maintained partial recovery even at 10 µg/ml, a concentration at which neutral-adapted strains failed to recover.

Across the Mtb strains, we did not observe consistent differences in susceptibility to isoniazid, rifampicin, or linezolid following acidic pH adaptations (Figs F and G in S1 Appendix), in contrast to the uniform response seen with ethambutol (Figs F and G in S1 Appendix). Rifampicin and linezolid are known to be effective against bacteria in nonreplicating states; therefore, we did not expect that an increased proportion of growth-arrested (e.g., nonreplicating) bacteria resulting from acidic adaptation would decrease susceptibility to these drugs. Acidic adaptation may not affect isoniazid treatment in the same way as ethambutol because acidic adaptation is thought to increase inhibition of the target of isoniazid, InhA, via PhoPR-dependent upregulation of NAD⁺ [19,32], thereby increasing isoniazid susceptibility via a mechanism independent of growth arrest. These findings suggest that acidic adaptation enhances Mtb tolerance to ethambutol, but not isoniazid, rifampicin, or linezolid.

The resazurin assay reports bulk-level metabolic states as a measure of survival of drug treatment; these dose-response assays do not distinguish between the relative survival of cells in the growing versus the growth-arrested subpopulations. We hypothesized that ethambutol tolerance arises primarily from the nongrowing cell subpopulation because ethambutol inhibits arabinogalactan synthesis, a process essential for cell wall elongation in actively growing cells [38,39]. To test this hypothesis, we conducted live-cell imaging to assess which cells (growing or nongrowing) resumed growth after drug removal (Fig 3C and 3D and S4 Movie). To identify individual cells as growing or nongrowing immediately preceding drug exposure, the CDC1551 WT strain was adapted to pH 5.9 for 4 days and labeled with HADA. Prior to the experiment, we confirmed that HADA labeling itself does not affect ethambutol susceptibility (Fig H in S1 Appendix). Cells were then loaded into a microfluidic device for time-lapse imaging, where they were treated with a high ethambutol dose (245 µg/ml) for 2 days and allowed to recover for 8 days in drug-free fresh media. The purpose of the live-cell imaging was to identify the ethambutol-tolerant cells; therefore, the high ethambutol dose was chosen to ensure complete elimination of susceptible cells, leaving only tolerant cells for analysis. The high dose was also necessary due to technical differences in environmental conditions between the microfluidic device and plate-based assays, such as the continuous flow of fresh, nutrient-rich media through the cells and the air permeability of the microfluidic device, which can enhance survival (details in Materials and methods).

We observed that nongrowing cells formed during acidic adaptation predominantly contributed to recovery after the ethambutol treatment (Fig 3D, Table A in S1 Appendix). Only 1% of previously growing cells resumed growth upon ethambutol removal (5 out of 736 cells), whereas 13% of the nongrowing cells recovered (20 out of 152 cells) (Table A in S1 Appendix). Together, these data suggest that nongrowing cells that arise after acidic adaptation have reduced ethambutol susceptibility.

### PhoPR is a partial regulator of growth and ethambutol tolerance in acidic adaptation

**Fig 4:**
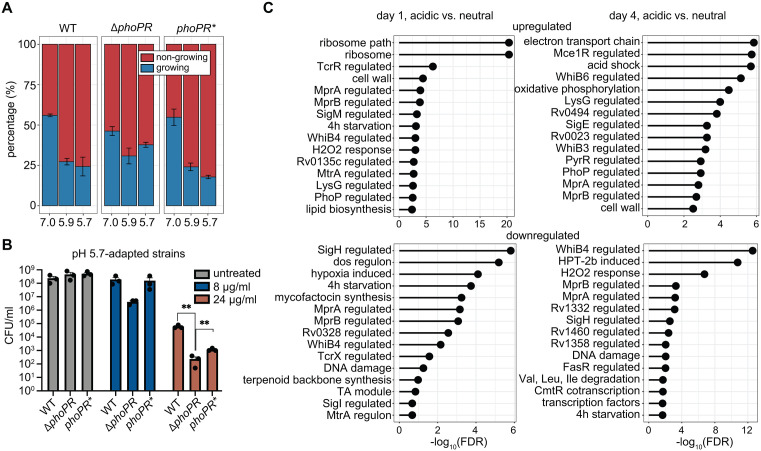
PhoPR is involved in growth arrest and ethambutol susceptibility under acidic conditions. **(A)** Proportions of growing (HADA-positive, blue) and nongrowing (HADA-negative, red) cells after 4-day adaptation to pH 7.0, 5.9, or 5.7. Three strains were used—CDC1551 streptomycin-resistant WT (STR-R), *phoPR* mutant, and complemented strains (details in Materials and methods). See Fig 1A for experimental design. Data are from biological triplicates. The data underlying this Figure can be found in https://doi.org/10.5281/zenodo.20438984. **(B)** After a 4-day adaptation at pH 5.7, cells were treated with ethambutol (untreated, 8, or 24 µg/ml) for 10 days, then plated for CFU. Biological triplicates; mean with SD. *p*-values between WT and mutant (*p* = 0.004), and mutant and complemented (*p* = 0.009); unpaired two-tailed *t* test. The data underlying this Figure can be found in S1 Data. **(C)** Multi-strain pathway and gene regulator enrichment analysis from RNA-seq of CDC1551, isolate 1, and isolate 2 after 1 or 4 days at neutral and acidic (pH 5.9). Data are from biological triplicates. Differentially expressed genes (DEGs) (adjusted *p*-values < 0.05), shared across all strains under acidic conditions, were analyzed to identify commonly regulated pathways by the upstream regulators (hypergeometric test). The readout represents the statistical significance of differential gene expression. FDR, false discovery rate. The data underlying this Figure can be found in https://doi.org/10.5281/zenodo.20438984 and the GEO repository.

PhoPR is a two-component regulatory system, and a key regulator of cellular adaptation to acidic conditions [32,40,41]. It is the best-characterized and best-known acid-responsive regulator in mycobacteria, modulating metabolism and growth under acidic conditions [19,20,32]. For example, at mildly acidic pH (around pH 5.7), PhoPR is induced and slows Mtb growth or drives growth arrest on many single-carbon sources [32,40,41]. We therefore focused on PhoPR to determine the extent to which it regulates nongrowing cell formation and ethambutol tolerance under acidic conditions. To this end, we performed fixed-cell imaging using FDAA labeling in CDC1551 WT, *phoPR*-deleted mutant (Δ*phoPR)*, and *phoPR*-complemented (phoPR*) strains at neutral (pH 7.0) and acidic conditions (pH 5.9 and 5.7) (Fig 4A). To construct PhoPR mutants, a CDC1551 strain with a streptomycin-resistant background was used (see Materials and methods) [40]. Due to the well-characterized genetic background, which enables a clear comparison between the wild-type and PhoPR mutant, we used the CDC1551 strain to investigate the role of PhoPR in nongrowing cell formation and ethambutol tolerance. Clinical isolates were not used to avoid potential strain-specific genetic factors and drug response phenotypes that could confound the effects on both growth arrest and drug susceptibility [42–45]. By observing consistent polar labeling across all imaged strains, including the *phoPR* mutant, we confirmed that HADA incorporation reflects growth rather than changes in permeability or peptidoglycan metabolism, which would alter labeling patterns (Fig I in S1 Appendix). Though increased nongrowing cell formation was observed at pH 5.9 compared to pH 7.0, the result was independent of PhoPR, as nongrowing cell proportions did not differ between WT and phoPR* strains compared to the Δ*phoPR* strain (Fig 4A). However, at pH 5.7, *phoPR*-dependent nongrowing cell formation was evident, with significantly fewer nongrowing cells in the Δ*phoPR* strain than in WT and complemented strains (*p*-value 0.004 and 0.009, respectively). These data suggest that the PhoPR regulon contributes to increasing the proportion of the growth-arrested subpopulation at pH 5.7.

We examined the role of PhoPR in ethambutol tolerance under acidic conditions by comparing survival among WT, Δ*phoPR* mutant, and phoPR* strains. Survival rate was measured by counting CFUs at pH 5.7 using these three strains and two ethambutol concentrations derived from Figs 3B and 4B. To compare the survival of acidic-adapted cells between wild-type and *phoPR*-deleted mutants, two ethambutol doses were selected to capture the point at which survival of acidic-adapted cells begins to decrease, allowing a direct comparison between strains. In the absence of ethambutol, a similar CFU/ml was observed among all strains. However, upon treatment with 24 µg/ml ethambutol, the Δ*phoPR* mutant exhibited significantly lower CFU counts than WT and phoPR* strains, suggesting that PhoPR contributes to the ethambutol tolerance observed in this context. Although a similar trend was observed at 8 µg/ml ethambutol, the difference was not statistically significant.

Because deletion of *phoPR* can influence cell wall lipid composition [46], we examined whether the increased ethambutol susceptibility of Δ*phoPR* was due to a change in membrane properties. We observed that Δ*phoPR* exhibited higher FM 4–64 staining when compared with WT and phoPR* at pH 5.7 and pH 7 (Fig J in S1 Appendix). Because there was no difference in ethambutol susceptibility between WT and Δ*phoPR* at pH 7.0 (Fig J in S1 Appendix), we concluded that the changes in membrane properties in Δ*phoPR* do not lead to increased ethambutol susceptibility. Together, these data suggest that the Δ*phoPR* Mtb is more sensitive to ethambutol than WT Mtb, not because of changed membrane properties, but because of an increase in the proportion of nongrowing cells.

### Multiple regulatory pathways are involved in Mtb acidic adaptation

Collectively, these findings demonstrate that although PhoPR is a key regulator of growth arrest and drug tolerance under acidic conditions (in pH 5.7), additional regulatory mechanisms are likely involved in this complex adaptation. To identify other possible regulatory mechanisms involved in the transition to growth arrest and ethambutol tolerance under acidic conditions compared to neutral conditions, we performed RNA sequencing (RNA-seq) on CDC1551 WT and two clinical isolates after four days of adaptation to neutral (pH 7.0) or acidic (pH 5.9) conditions. To determine pH-specific transcriptional changes common to all strains, we subjected the intersection of their DEGs to pathway and gene regulatory program enrichment analysis. We found that PhoP-regulated genes were one of the most DEGs in acidic conditions across all tested strains (Fig 4C, adjusted *p*-value = 3.1 × 10^−3^). However, other regulatory pathways were also upregulated, suggesting that while PhoPR plays a key role, it may not be the sole master regulator of growth control in acidic environments. Specifically, other transcriptional programs known to modulate growth and division, and more enriched than the PhoP-regulated gene sets, included MprAB-, WhiB4-, WhiB3-, MtrA-, TcrX-, and SigH-regulated pathways (Fig 4C). The genes regulated by the MprAB two-component system were significantly upregulated at both day 1 and day 4 of adaptation (mean adjusted *p*-value = 1.3 x 10^4^ and 1.83 x 10^3^, Fig 4C). MprAB responds to pH and cell envelope stress and coordinates the expression of over 200 genes that control resistance to cell envelope damage and growth speed. The MprAB system may thus play a key role in generating a nongrowing cell subpopulation in acidic conditions that is tolerant to ethambutol-induced cell wall damage.

## Discussion

The ability of Mtb to adapt to different environments during infection, including to acidic pH, is critical to Mtb survival both in the host and during drug treatment, yet we lack an understanding of how individual cells adapt to these environments. Here, using single-cell approaches, we find that individual bacteria adapt to acidic pH conditions in a manner that could not be inferred from population-level measurement alone. We find that Mtb adapts to acidic pH not by uniformly slowing growth, but by increasing the proportion of cells in the nongrowing subpopulation. We observed a significant proportion of nongrowing cells not only under acidic pH conditions but also in neutral conditions. These data suggest that nongrowth is not a rare state but rather a common state for Mtb even in nutrient-rich conditions with limited environmental stress. The propensity to enter this nongrowing state was higher in recently isolated clinical strains compared to those of lab-adapted strains. Clinical isolates have had fewer passages in laboratory growth conditions. Therefore, we expect they would retain adaptations from the human host, thereby contributing to phenotypic plasticity. Mtb are known to enter nongrowing states under severe stressors, such as hypoxia, or in lipid-rich conditions of the caseum. But our data demonstrate that there is a high proportion of nongrowing cells emerging after short adaptations even to rich, mild conditions in the laboratory, suggesting that a growth-arrested state may be more typical for Mtb than previously thought.

We found that the bulk slowed growth at acidic pH is due to an increased proportion of the nongrowing cell subpopulation, not to slower single-cell growth rates (Figs 2C and 2D and E in S1 Appendix). Correspondingly, cells that continued to grow throughout the experiment in the acidic condition maintained the same growth rate as those in the neutral condition (Fig 2E). These data show that Mtb tunes its growth rate not by uniformly slowing or accelerating single-cell growth speeds, but instead by tuning the proportion of growth-arrested cells in the population; i.e., cells do not slightly change their growth rates at the single-cell level but shift from a regular growth mode to a growth-arrested mode. Maintaining this heterogeneous population of cells likely provides a survival benefit in the face of various stressors (bet-hedging) [11,47–49].

We found that the growth-arrested subpopulation in the acidic growth condition was more tolerant to ethambutol treatment than the growing subpopulation (Fig 3B and 3D). Antibiotic efficacy is known to be strongly dependent on the physiologic state of the bacteria [50], and previous studies in mycobacteria have confirmed this on the single-cell level [23,24,26,51]. In this study, the acid-adapted Mtb that were growth-arrested were protected against ethambutol across different strains. Protection of the growth-arrested subpopulation did not generalize to other antibiotics tested (Figs F and G in S1 Appendix), demonstrating that the physiologic state of the nongrowing cells may be protective against some but not all types of stressors. Future studies may examine whether growing and nongrowing subpopulations in acidic conditions are differentially susceptible to host-relevant stresses, such as reactive nitrogen species, reactive oxygen species, or hypoxia. Additionally, we observed that, in clinical isolates, neutral-adapted cells remained less tolerant to ethambutol despite a high proportion of nongrowing cells under neutral pH conditions. Therefore, while growth arrest contributes to ethambutol tolerances, other mechanisms, such as gene evolution in response to drug-linked pressure [44], likely play a role and should be investigated in future studies.

We did not examine the mechanisms by which Mtb exposed to acid stress better survives ethambutol treatment, but it is possible that changes in cell wall-related gene expression play a major role. A gene *embB*, which encodes a protein involved in the biosynthesis of arabinogalactan of the cell wall, mediates resistance to ethambutol treatment [52–54] and was significantly upregulated in acidic conditions compared to neutral conditions in the three tested strains, which included two clinical strains (Table B in S1 Appendix). Future studies may examine whether the upregulation of *embB* directly contributes to the higher survival of Mtb in acidic conditions.

Although both isoniazid and ethambutol target the mycobacterial cell wall, their effects on bacterial survival after acidic adaptation diverged due to their distinct mechanisms of action. Isoniazid is a prodrug that is activated by the catalase-peroxidase KatG, producing isonicotinoyl radicals that bind NAD⁺ to form isonicotinoyl-NAD [55,56]. Isonicotinoyl-NAD inhibits InhA, a key enzyme involved in mycolic acid biosynthesis, thereby compromising cell wall integrity [57,58]. Under isoniazid pressure, Mtb downregulates genes encoding NADH dehydrogenase to limit NAD⁺ production, reducing isonicotinoyl-NAD formation and mitigating InhA inhibition to promote survival [59–61]. However, under acidic conditions, PhoPR regulation enhances NAD⁺ production, leading to increased isonicotinoyl-NAD formation and increased bacterial susceptibility to isoniazid through enhanced InhA inhibition [62–64]. These findings underscore that drugs with similar targets, such as ethambutol and isoniazid, can yield distinct outcomes under host-relevant stress conditions due to differences in their activation pathways.

PhoPR is a master regulator of Mtb’s transcriptional response to acidic conditions [14,19,32,40,41,65], but we found that it was only partially responsible for controlling the proportion of the nongrowing cell subpopulation in response to pH fluctuation (Fig 4A). A recent study suggests that PhoPR integrates multiple environmental signals, including carbon dioxide levels, as well as acidic pH, to modulate transcriptional responses [41]. To explore additional regulatory systems beyond PhoPR, we examined transcriptional changes associated with acidic adaptation across three strains. The most significantly upregulated pathway between neutral and acidic conditions common to all strains tested was the ribosome biogenesis, potentially reflecting a compensatory mechanism to counteract reduced protein synthesis rates caused by acid stress [66,67]. While PhoP-regulated genes were significantly differentially expressed, other regulators involved in sensing pH, altering growth and division, and resisting cell wall stress were more strongly enriched (Fig 4C). For example, MprAB is known to respond to pH and controls the expression of genes that confer resistance to cell wall damage [68]. The MtrAB two-component system is also known to play a crucial role in dormancy, cell division, cell wall metabolism, and modulating drug susceptibility, and MtrA-regulated genes are significantly differentially expressed at acidic pH [69–74]. More comprehensive genetic analysis may find that MprAB, WhiB4, SigH, or other differentially expressed regulons play a larger role than PhoPR in generating the nongrowing cell subpopulation. It may be that many transcriptional regulators are involved to provide redundancy and robustness to the acid stress response in Mtb. A recent study found that several transcription regulators, including PhoPR, exhibit genetic variations in clinical isolates that are associated with drug response [44]. It is also likely that some regulators integrate multiple cues to regulate physiological responses; for example, Mce3R integrates cues from carbon sources and potassium [75]. Simultaneous integration of multiple environmental cues may allow Mtb to fine-tune its transcriptional response. Another explanation for why so many transcriptional regulators are involved in responding to acid stress could be that early and delayed responses require different regulation. In support of this, genes regulated by the redox-dependent WhiB4 regulator are upregulated on day one but downregulated by day four (Fig 4C) [76].

In addition to transcriptional regulation, carbon source availability can drive metabolic shifts that influence bulk slow growth or growth arrest under acidic conditions [18–21]. For example, Mtb fully arrests growth at mildly acidic pH (pH 5.7) when provided with glucose, glycerol, or most TCA cycle intermediates as sole carbon sources [20]. In contrast, Mtb can maintain growth at pH 5.7 when utilizing alternative carbon sources such as cholesterol, acetate, and oxaloacetate [20,21,75]. In this study, Mtb strains were grown in 7H9 medium supplemented with OADC (oleic acid, albumin, dextrose, and catalase), a rich carbon source mixture. Therefore, a limitation of our study is that we did not assess growth patterns under conditions using individual carbon sources.

Many pathogens, such as *Streptococcus, Escherichia, Salmonella*, and *Pseudomonas*, have adapted mechanisms to survive at acidic pH. These include the activation of acid resistance systems and the downregulation of H^+^ pumps to limit proton entry into the cell [77,78]. In macrophage environments, subpopulations of *Salmonella* are known to slow their growth while maintaining an active metabolism [79]. It may be that Mtb and *Salmonella* similarly induce growth arrest in a subpopulation while another subpopulation continues to grow at the same rate as a bet-hedging strategy [79]. Future studies may investigate whether other pathogens coordinate bifurcations of growth and how this mechanism impacts bacterial fitness.

Together, our study highlights how single-cell approaches can provide key insights that may be overlooked by population-level measurements alone. By understanding the behaviors and variations of individual cells, we can investigate how different subpopulations arise and respond to host- and environmental-stressors. Future studies may also aim to identify the genetic and metabolic regulators that control the growth-state decisions in individual cells, as these factors may serve as critical targets for therapeutic intervention.

## Materials and methods

### Mtb strains

Six Mtb strains were used in this study: CDC1551 and H37Rv and four clinical strains, 24TB069, 24TB041, 24TB25, and DTU117 from [25] that are referred to as isolates 1–4, respectively. These strains were selected to reflect the diversity of Mtb genetics and virulence present in TB patients in Vietnam. The strains encompass all three major Mtb lineages found in TB patients in Vietnam: 24TB069, Indo-Oceanic; 24TB041, East-Asian; 24TB25, East-Asian; and DTU117, Euro-American. They were also selected to represent a range of degrees of virulence in a macrophage lysis model: 24TB069, moderate; 24TB041, high; 24TB25, high; and DTU117, high. The CDC1551 Δ*phoPR*, and phoPR* (complemented mutant) strains have been previously described [40]. Briefly, a *phoPR* mutant was generated using flanking-region cloning into either side of the hygromycin cassette and transformed into a streptomycin-resistant *rpsL* CDC1551 strain. Complementation (phoPR*) was achieved by integrating the *phoPR* gene with its native promoter into the *attB* site via the integrative vector pMV306.

### Bacterial culture

Mtb strains were grown in standard medium consisting of 7H9 broth (ThermoFisher; DF0713-17-9) with 0.05% Tween 80 (ThermoFisher; BP338-500), 0.2% glycerol (ThermoFisher; G33-1), and 10% Middlebrook OADC (ThermoFisher; B12351). Strains were grown to OD_600_ of 0.5–1.0 from frozen at 37 °C with mild agitation (100 rpm). To assess the effect of the initial growth phase on growth arrest after pH adaptation, cells were grown to OD_600_ of 0.1, 0.5, and 1 for comparison. They were then back-diluted to OD_600_ 0.05 and grown to mid-log phase (OD_600_ 0.5–1.0) before experimental use.

### Fluorescent staining

The fluorescent D-amino acids (FDAA) RADA (Tocris; 6649) and HADA (Tocris; 6647) were used in this study. FDAA powder was dissolved in DMSO to a stock concentration of 100 mM and stored short-term at −80 ºC. Cells were incubated in 25 µM RADA or HADA for 6 h before fixation at each time point. Heat-killed CDC1551 cells were used as a negative control. For live-cell imaging, cells were incubated in 100 µM HADA for either 12 or 24 h prior to the start of imaging. For the permeability assay, fixed Mtb were stained with a solution of 5.2 µg/mL of FM4−64FX (ThermoFisher; F34653), PBS (ThermoFisher; 20012-027), and PBST. Cells were incubated in the presence of dye at room temperature in the dark for 30 min. Stained cells were washed once with 150 µL of PBST prior to imaging.

### Fixation of Mtb cells

For the experiments used for fixation, once a cell culture entered mid-log phase (see “Bacterial culture” section), strains were passaged to OD_600_ 0.05 in three different media types: standard 7H9 pH 7.0, pH 6.2, and pH 5.9. pH-adjusted 7H9 media were made with 100 mM MOPS buffer (SigmaAldrich; M3183) added to standard medium and NaOH to adjust pH to 7.0, or 100 mM MES buffer (SigmaAldrich; M2933) added to standard medium and NaOH to adjust the pH to 6.2 or 5.9. Each culture was placed on 96-well plates (150 µl/well) and incubated at 37 °C in humidified bags until fixation. Three biological replicates were generated for each strain and each condition.

At designated time points, Mtb cultures were fixed in paraformaldehyde (Alfa Aesar, 43368) at a final concentration of 4% for 1 h, and removed from the biosafety level-3 facility. After fixation, the samples were washed twice with PBST and then resuspended in PBST. Plates were sealed (ThermoFisher optically clear plate seals; AB1170), and all samples were stored at 4 ºC until imaging.

### Fixed-cell imaging

Fixed Mtb cells were spotted onto 1% agarose pads (SigmaAldrich; A3643-25G) as in [80]. In Fig 1, images were captured with a widefield DeltaVision PersonalDV (Applied Precisions) microscope. Bacteria were illuminated using an InsightSSI Solid State Illumination system with transmitted light for brightfield microscopy. RADA was visualized with Ex. 544 nm and Em. 570 nm wavelengths. Montage images were generated using a custom macro that captures 25 individual fields of view per image. Images were recorded with a DV Elite CMOS camera for all channels. For imaging of the WT, Δ*phoPR*, and phoPR* strains (Fig 4), a widefield Zeiss Axio Observer 7 with Definite Focus microscope and Hamamatsu ORCA-Fusion Digital CMOS camera was used. Bacteria were illuminated with phase contrast, and HADA was visualized with Ex. 370–400 nm and Em. 410–440 nm using the 90 HE DAPI reflector and the LED-Module 385 nm light source. FM 4–64 stained Mtb were imaged with Ex. 489–533 nm and Em. 542–832 nm using the 91 HE filter set and the LED-Module 511 nm light source.

### Fixed-cell image segmentation

Before image segmentation, the ImageJ plugin BaSiC was used to ensure an even distribution of illumination over all channels across the image [81]. Image segmentation and feature extraction were performed using a custom pipeline that relies on ilastik (v1.4.0) and CellProfiler (v4.2.1). Briefly, an ilastik pixel classifier was trained to distinguish between cell and background pixels and applied to all images. Using these pixel prediction maps, an ilastik object classifier was trained to discriminate single cells from clumps of cells or debris in the images. FDAA-positive regions were segmented using a separate ilastik pixel classifier trained to distinguish between fluorescent and background pixels. The resulting cell boundary predictions and regions of FDAA positivity were used as input for a CellProfiler pipeline. These two ilastik prediction sets were converted to objects in CellProfiler using the “IdentifyPrimaryObjects” module with manual thresholding, and the “RelateObjects” module was used to relate the FDAA-positive regions to each segmented cell. The single-cell measurements were exported in CSV format using the “ExportToSpreadsheet” module for downstream analysis and plotting.

### Live-cell microscopy

Strain CDC1551 was used for live-cell imaging. Prior to live-cell imaging experiments, Mtb cells were stained with 100 µM of HADA in 10 ml standard 7H9 broth media for 24 h. Cells were then washed twice with phosphate-buffered saline with 0.2% Tween-80 (PBST) and resuspended in pH 7.0 or pH 5.9-adjusted 7H9 media.

Before loading Mtb cells into a custom polydimethylsiloxane (PDMS) microfluidic device, cells were filtered through a 10 µM membrane filter to remove clumps. Mtb cells were then loaded into a microfluidic device, as in [24]. The device contained a main microfluidic feeding channel with a height of 10–17 µm and viewing chambers with a diameter of 60 µm and a height of 0.8–0.9 µm. Fresh medium was delivered to the cells at 5 µl/min using a microfluidic syringe pump. The device was placed on an automated microscope stage within an environmental chamber maintained at 37 ºC. Mtb cells were imaged for 120 h at 1-h intervals using a widefield Deltavision PersonalDV (Applied Precision) microscope. Cells were illuminated with an InsightSSI Solid State Illumination system every hour. Cells were imaged using transmitted light brightfield microscopy, and HADA was visualized with 425–445 nm excitation and 460–510 nm emission wavelengths.

#### Live-cell imaging with ethambutol.

For ethambutol-treated live-cell imaging, the CDC1551 WT strain was used. Prior to imaging, the cells were pre-adapted to pH 5.9 7H9 media for four days. HADA (100 µM) was added during the last 12 h of the adaptation period to mark whether cells were growing (HADA-positive) or nongrowing (HADA-negative) after adaptation. For the pH 5.9 media that was flowed in the microfluidic device, media conditioned using the TB auxotroph strain adapted to pH 5.9 7H9 media was mixed 1:1 with pH-adjusted regular 7H9 media [30,82]. Once the cells were loaded into the device, the pH-adjusted media containing 245 µg/ml of ethambutol was delivered through the device. For the negative control, 1% DMSO was delivered instead of ethambutol. After 2 days, the syringe containing ethambutol or 1% DMSO was replaced with a new syringe containing fresh, unbuffered media without ethambutol for the recovery period. For this unbuffered media, unbuffered spent media was mixed 1:1 with regular unbuffered 7H9 media. The recovery period was imaged for eight days. The cells were imaged with bright field (32% transmission, 0.1 s exposure time) and CFP filter (5% transmission, 0.1 s exposure time).

### Live-cell image annotation

Before segmentation, each channel was merged into one image. ImageJ plugin ObjectJ was used to hand-annotate cell length and growth throughout live-cell imaging. Each annotated movie was then extracted as a CSV file for further analysis.

### Segmented data analysis

Among the annotated cells, those that could not be determined to be either growing cells or cells that converted from growing to nongrowing were excluded from the analysis. This includes cells that were born late in the movie or that could no longer be annotated due to clump formation or overlap with other cells due to high cell density. The annotation in each frame was extracted, containing information on cell length and growth at each pole over time (1-h timescale). The ObjectJ data were exported to an XML file, then converted to a CSV file. For the single-cell analysis, we used custom scripts in MATLAB that calculated and collated single-cell data: length at birth and division, growth from each pole, and interdivision time. To classify cells as nongrowing, growing, or growing to nongrowing, we used a combination of quantitative and visual annotation. First, we smoothed hourly growth rates using a low-pass filter with coefficients equal to 5 using the “smooth” function in MATLAB. Cells whose smoothed growth rate throughout the movie was close to 0 dL/dT were classified as nongrowing. Cells whose smoothed growth rate was > 0/dL/dT and fitted a linear regression with low error, *R*-squared > 0.9, were classified as growing. If the fit was < 0.9, then the cells were classified as “growing to nongrowing,” since a poor fit indicates the cell growth did not follow a linear trajectory and must have slowed. Accurate binning into these categories was confirmed by plotting cell length over time and visually inspecting the slopes.

### Growth rate analysis from live-cell imaging

To compute the instantaneous growth rate of Mtb cells, we measured cell lengths hourly throughout the experiment. To account for cell length variation caused by instability in the microscopy field of view, we computed a smoothed instantaneous growth rate using a 20-h rolling window. For the simulated null model data, instantaneous growth rates were generated by replacing the experimental data with randomly sampled values so that the final population-averaged growth rate matched that of the experimental data. For the neutral pH simulated data, these values were sampled from a distribution with a mean equal to that of the neutral pH experimental growth rate, mean = 0.058 dL/h, and S.D. = 0.08 dL/h. For the acidic pH simulated data, we input values sampled from a distribution equal to the neutral pH simulated values (mean = 0.058 dL/h and S.D. = 0.08 dL/h) for the first 48 h. After 48 h of experiment time, the values were randomly sampled from a distribution of mean = 0.04 dL/h and S.D. = 0.02 dL/h, to reflect the null hypothesis that single-cell growth should be slowing but not halting. Again, these values were chosen so that the acidic pH simulated data’s population-averaged growth rate matched the 0.049 dL/h mean of the experimental data. The resulting dL/h measurements were smoothed with a 20-h rolling window in the same way as the experimental data.

### Preparation of charcoal agar plates

Charcoal agar plates were prepared as previously described [83]. Briefly, 450 ml of Middlebrook 7H10 agar containing 0.2% glycerol and 0.4% of activated charcoal was autoclaved in a flask. Once the autoclaved agar cooled to 55–65 ºC, the flask was placed on a magnetic stirrer hot plate to maintain the charcoal in suspension. Ten percentage Middlebrook OADC (ThermoFisher; B12351) was added. After pouring the 7H10-OADC-charcoal into a reservoir, 200 µl of 7H10-OADC-charcoal was transferred to each well of a 96-well plate using a 12-multichannel pipette. Once the poured agar solidified, the plates were placed in plastic bags and stored at 4 ºC until use.

### Resazurin assay after antibiotic treatment

Strains were adapted to their respective pH-buffered media for four days prior to antibiotic treatment. Cells were then back-diluted to OD_600_ 0.05 and treated with various drug concentrations in 384-well flat clear-bottom plates. Cells without drugs were used as a control. After 10 days of drug treatment at 37 ºC incubation, 10 µl of the cell culture was transferred into a new 96-well plate containing 200 µl of 7H10-OADC-charcoal per well to allow the charcoal to sequester the drugs used to treat Mtb [84]. After 10 days of recovery, 40 µl of sterile 1xPBST (0.05% Tween-80 in PBS) was added to prevent the dried charcoal from absorbing resazurin. 50 µl of freshly prepared resazurin solution—0.01% of resazurin in 5% Tween-80 in PBS, filtered—was added to each well. Then the plates were rocked back and forth a few times, placed in a plastic bag and a secondary container, and incubated at 37 ºC for 1 h. The fluorescence was read using a Biotek Synergy Neo2 Hybrid Multi-Mode Reader, with excitation at 530 nm and emission at 590 nm.

For the resazurin assay used to assess the effect of HADA labeling on ethambutol susceptibility, the same experimental design was used with CDC1551 cells, comparing HADA-labeled and unlabeled cultures.

### CFU assay for drug treatment

CDC1551 WT (STR-R), Δ*phoPR*, and phoPR* strains were used for the assay. Strains were first adapted to either pH 7.0 or pH 5.9 for 4 days prior to antibiotic treatment. After 4 days of pH adaptation, cells were back-diluted to OD_600_ 0.05 using each pH-adjusted medium and treated with ethambutol at 8 or 24 µg/ml. Untreated samples served as controls. After 10 days of ethambutol treatment, cells were washed twice with unbuffered 7H9 media for 10 min at 2,500 rpm. The cells were resuspended in unbuffered 7H9 media, serially diluted, and plated on 7H10 agar plates. The colony counting was performed after 3 weeks. The assay was performed using biological triplicate.

### Sample preparation for RNA sequencing

Biological triplicates of CDC1551, V1, and V3 strains were used for RNA sequencing. When the cells were at mid-log phase (OD_600_ 0.3–0.5) in the unbuffered media, the cells were back-diluted to OD_600_ 0.05 in 10 ml of buffered media (pH 7.0 or pH 5.9). The cells went through adaptation to each pH-adjusted medium, and were then harvested by centrifugation after either 1 or 4 days of adaptation. At each time point, GTC buffer was added to halt transcriptional response during RNA extraction, and the cells were washed twice. The cell pellets were resuspended in ~1 ml of GTC buffer (4M GTC + 0.5% sarcosyl + 25 mM sodium citrate + 0.1M β-mercaptoethanol) and stored at −80 °C at least overnight.

The RNA extraction protocol previously described was used [85]. On the day of extraction, the frozen GTC-treated pellets were thawed at 37 ºC, harvested by centrifugation, resuspended in pre-warmed triazole, and transferred to 2 ml O-ring tubes containing sterile 0.2 mm zirconia beads. The triazole mixture was then bead-beaten twice with an intensity of 6 meters per second for 45 s, followed by the addition of chloroform and vigorous shaking for 1 min. The samples were then centrifuged, and the aqueous layer was removed and placed in Qiagen RNeasy columns for RNA cleanup. Genomic DNA was removed from all samples by performing a DNase digest on the columns before elution in pre-warmed RNase-free water. The extracted RNA was then removed from the BSL-3 facility. Concentration and quality of RNA (260 nm/280 nm absorbance) were assessed using a nanodrop spectrophotometer; samples were then stored at −80 °C until submission. Samples were submitted to the Microbial ‘Omics Core (MOCP) and Genomics Platform at the Broad Institute of MIT and Harvard. QC, library preparation, ribodepletion, and paired-end sequencing on the Illumina NovaSeq 6000 following a modified RNAtag-Seq method [86] were performed by the MOCP.

### RNA-seq data processing and analysis

Sequencing reads were aligned to the *Mycobacterium tuberculosis* strain. Erdman = ATCC 35801 (RefSeq assembly accession: GCF_000350205.1) using Geneious Prime software (version 2023.2.1). The Geneious Prime software was used for the gene-level counts, and DESeq2 was used to identify genes that were differentially expressed in the acidic and neutral conditions. To identify acidic condition-responsive genes that were similarly differentially expressed across each strain, the up- and down-regulated gene lists were filtered by the criterion *p* < 0.05. Genes that were significantly differentially expressed and were regulated in the same direction (up or down) between all three strains were submitted for pathway and gene regulator enrichment analysis. To quantify enrichment *p*-values, a Fisher’s exact test was performed in R using fisher.test and a custom pathway and gene regulator annotation file that was curated through literature searches.

## Supporting information

S1 Appendix Figures A to J and Tables A to B. **Fig A**. FDAA labeling of growing Mtb cells with heat-killed control. CDC1551 cell cultures were either live or heat-killed and then treated with 25 µM HADA or RADA. HADA- or RADA-positive cells indicate actively growing cells. Scale bar, 2 µM. **Fig B.** Viability of nongrowing Mtb cells after acidic adaptation assessed by time-lapse imaging. CDC1551 cell cultures underwent 4 days of acidic adaptation, including 12 h of HADA labeling at the end of the adaptation period. The cells were then treated with 1% DMSO for 2 days under acidic conditions, followed by an 8-day recovery period in unbuffered media, during which time-lapse imaging was performed. Cells were divided into two groups: (i) HADA-positive (cells that were growing after acidic adaptation, *n* = 361) and (ii) HADA-negative (cells that were nongrowing after acidic adaptation, i = 166). The cells that resumed growth during the recovery period were counted, and the percentage of regrowing cells in each group was calculated. **Fig C.** Proportion of growing and nongrowing cells across different starting ODs after pH adaptation. CDC1551 cells were back-diluted into fresh media at an OD_600_ of 0.1, 0.5, or 1.0, and then grown to mid-log phase (OD 0.5–1.0) prior to pH adaptation. The proportions of RADA-negative (nongrowing) and RADA-positive (growing) cells are shown after four days of adaptation to neutral (pH 7.0) condition. Three biological replicates, error bars indicate the SD. The data underlying this Figure can be found in https://doi.org/10.5281/zenodo.20438984. **Fig D.** Proportion of growing and nongrowing cells across six strains after pH adaptation. The proportions of RADA-negative (nongrowing) and RADA-positive (growing) cells are shown after one or four days of adaptation to neutral (pH 7.0) and acidic (pH 6.2 and pH 5.9) conditions. Among the six strains, two are Mtb laboratory strains (CDC1551 and H37Rv), and the other four are more recent clinical isolates (*n* = 3 biological replicates, error bars indicate the SD). Significance was measured with one-way ANOVA and Dunnett‘s post-hoc test against neutral pH: ns, *p* ≥ 0.05, *p < 0.05, **p < 0.01, ***p < 0.001. The data underlying this Figure can be found in https://doi.org/10.5281/zenodo.20438984. **Fig E.** Slowed growth of Mtb at low pH is controlled by bimodal rate tuning. (A) Density (OD_600_) of CDC1551 strain cultures adapted to a range of neutral and acidic pH levels. p values, pH 7.0 versus pH 5.9, *p* = 0.0014; pH 5.9 versus pH 5.7, *p* = 0.18; pH 5.7 versus pH 5.5, *p* = 0.019 (*n* = 3 biological replicates, error bars indicate the SD). (**B**) Kernel density estimate of the growth rates (change in length per hour) of Mtb cells across 5 days of neutral (green, *n* = 248) or acidic (orange, *n* = 135) adaptation. The data underlying this Figure can be found in S1 Data (A) and https://doi.org/10.5281/zenodo.20438984 (B). **Fig F.** Survival of Mtb adapted to acidic or neutral conditions after 2-day treatment with TB antibiotics. Four strains were used in the resazurin assay, including two laboratory strains and two clinical isolates. Ethambutol (EMB), isoniazid (INH), rifampicin (RIF), and linezolid (LZD) were added at various concentrations. Cells underwent 10 days of drug treatment followed by 10 days of recovery after drug removal. Following the recovery period, resazurin was added and incubated for 1 hour, after which fluorescence was measured to assess viability. Green indicates the neutral-adapted strain, and orange indicates the acidic-adapted strain. The resazurin fluorescence levels at the two pH values were normalized, with untreated strains set to 100%. Data are presented as the mean with SD (*n* = 3 biological replicates). The data underlying this Figure can be found in S1 Data. **Fig G.** Survival of Mtb adapted to acidic or neutral conditions after 10-day treatment with TB antibiotics. Four strains were used in the resazurin assay, including two laboratory strains and two clinical isolates. Ethambutol (EMB), isoniazid (INH), rifampicin (RIF), and linezolid (LZD) were added at various concentrations. Cells underwent 10 days of drug treatment followed by 10 days of recovery after drug removal. Following the recovery period, resazurin was added and incubated for 1 h, after which fluorescence was measured to assess viability. Green indicates the neutral-adapted strain, and orange indicates the acidic-adapted strain. The resazurin fluorescence levels at the two pH values were normalized, with untreated strains set to 100%. Data are presented as the mean with SD (*n* = 3 biological replicates). The data underlying this Figure can be found in S1 Data. **Fig H.** Effect of HADA labeling on ethambutol susceptibility in CDC1551 strain. Resazurin assay was performed following the same experimental design as in Fig 3A, using unbuffered media. HADA-labeled and unlabeled cells were treated with various doses of ethambutol, and resazurin fluorescence was measured to assess ethambutol susceptibility. The green line represents HADA-labeled cells, and the gray line represents unlabeled cells. Data are from three biological replicates, and error bars indicate the SD. The data underlying this Figure can be found in S1 Data. **Fig I.** FDAA-labeled Mtb cells, including wild type, *phoPR* mutant, and *phoPR*-complemented strains. Acidic-adapted CDC1551 wild type, phoPR-deleted mutant, and phoPR-complemented cells were labeled with 25 µM RADA and imaged. The red region indicates RADA labeling. **Fig J.** Deletion of PhoPR increases cell permeability at both neutral and acidic pH. The CDC1551 WT, Δ*phoPR*, and phoPR* strains were adapted to pH 7, pH 5.9, and pH 5.7 for one day and subsequently fixed and stained with FM 4–64 fluorescent dye. Mean and standard error are shown (*n* = 3 biological replicates). Significance was assessed with a one-way ANOVA and Tukey’s post-hoc test: ns *p* ≥ 0.05, *p < 0.05, **p < 0.01, ***p < 0.001, ****p < 0.0001. The data underlying this Figure can be found in https://doi.org/10.5281/zenodo.20438984. **Table A.** Recovery quantification of ethambutol-treated Mtb. Growth was determined based on HADA positivity (growing) or negativity (nongrowing), and recovery was marked by an increase in cell length during the drug-free recovery period. Results are from one experiment (Fisher’s exact test *p*-value = 3.8 × 10^−12^). **Table B.** pH-responsive genes that synergize with ethambutol when knocked down. The intersection of the top 500 differentially expressed genes across all 3 strains was sorted according to their fitness decrease (log2 fold change) upon CRISPRi knock down [87]. The top 10 genes are displayed.
(PDF)

S1 Movie A video of acidic (pH 5.9) adapted Mtb CDC1551 recovering after 2-day 1% DMSO (untreated control) treatment; cells were FDAA-labeled after 4 days of acidic adaptation to mark growth status. (MOV)

S2 Movie A video of the FDAA pulse-labeled Mtb CDC1551 wild-type strain in neutral (pH 7.0) standard growth medium supplemented with Middlebrook 7H9. (MOV)

S3 Movie A video of the FDAA pulse-labeled Mtb CDC1551 wild-type strain in acidic (pH 5.9) standard growth medium supplemented with Middlebrook 7H9. (MOV)

S4 Movie A video of acidic (pH 5.9) adapted Mtb CDC1551 recovering after 2-day ethambutol treatment; cells were FDAA-labeled after 4 days of acidic adaptation to mark growth status. (MOV)

S1 Data Processed time-lapse data of FDAA pulse-labeled Mtb CDC1551 wild-type strain in neutral (pH 7.0) standard growth medium supplemented with Middlebrook 7H9. (CSV)

S2 Data Processed time-lapse data of FDAA pulse-labeled Mtb CDC1551 wild-type strain in acidic (pH 5.9) standard growth medium supplemented with Middlebrook 7H9. (CSV)

S3 Data Growth speed calculated from time-lapse data of FDAA pulse-labeled Mtb CDC1551 wild-type strain in neutral (pH 7.0) standard growth medium supplemented with Middlebrook 7H9. (CSV)

S4 Data Growth speed calculated from time-lapse data of FDAA pulse-labeled Mtb CDC1551 wild-type strain in acidic (pH 5.9) standard growth medium supplemented with Middlebrook 7H9. (CSV)

S5 Data Numerical data underlying the graphs presented in the figures. (XLSX)

## Abbreviations

CFU: colony-forming unit
DEGs: differentially expressed genes
FDAA: fluorescent D-amino acid
Mtb: *Mycobacterium tuberculosis*
PBST: phosphate-buffered saline with 0.2% Tween-80
PDMS: polydimethylsiloxane
RNA-seq: RNA sequencing
TB: Tuberculosis.
