# Supplementary material for: Growth arrest of Mycobacterium tuberculosis in acidic environments enhances their survival of antibiotic treatment
Source: PLoS Biol. 2026 Jun 22;24(6):e3003857. doi: 10.1371/journal.pbio.3003857 (PMC13298992; doi:10.1371/journal.pbio.3003857)
Supplement: S1 Appendix — Fig A. FDAA labeling of growing Mtb cells with heat-killed control. CDC1551 cell cultures were either live or heat-killed and then treated with 25 µM HADA or RADA. HADA- or RADA-positive cells indicate actively growing cells. Scale bar, 2 µM. Fig B. Viability of nongrowing Mtb cells after acidic adaptation assessed by time-lapse imaging. CDC1551 cell cultures underwent 4 days of acidic adaptation, including 12 h of HADA labeling at the end of the adaptation period. The cells were then treated with 1% DMSO for 2 days under acidic conditions, followed by an 8-day recovery period in unbuffered media, during which time-lapse imaging was performed. Cells were divided into two groups: (i) HADA-positive (cells that were growing after acidic adaptation, n = 361) and (ii) HADA-negative (cells that were nongrowing after acidic adaptation, i = 166). The cells that resumed growth during the recovery period were counted, and the percentage of regrowing cells in each group was calculated. Fig C. Proportion of growing and nongrowing cells across different starting ODs after pH adaptation. CDC1551 cells were back-diluted into fresh media at an OD600 of 0.1, 0.5, or 1.0, and then grown to mid-log phase (OD 0.5–1.0) prior to pH adaptation. The proportions of RADA-negative (nongrowing) and RADA-positive (growing) cells are shown after four days of adaptation to neutral (pH 7.0) condition. Three biological replicates, error bars indicate the SD. The data underlying this Figure can be found in https://doi.org/10.5281/zenodo.20438984. Fig D. Proportion of growing and nongrowing cells across six strains after pH adaptation. The proportions of RADA-negative (nongrowing) and RADA-positive (growing) cells are shown after one or four days of adaptation to neutral (pH 7.0) and acidic (pH 6.2 and pH 5.9) conditions. Among the six strains, two are Mtb laboratory strains (CDC1551 and H37Rv), and the other four are more recent clinical isolates (n = 3 biological replicates, error bars indicate the [file pbio.3003857.s001.pdf]

S1 Appendix

Eun Seon Chung†, William C. Johnson†, Maliwan Kamkaew, Timothy A. Fitzgerald, Morgan E. McNellis, Trever C. Smith II, Srinivasan Vijay, Nguyen Thuy Thuong Thuong, Shumin Tan, Bree B. Aldridge*

†These authors contributed equally to this work
*Corresponding author: Bree Aldridge
Email: bree.aldridge@tufts.edu

This PDF file includes:

Figs A to J
Tables A to B

Other supporting materials for this manuscript include the following:

Movies S1 to S4
Data S1 to S5

Fig A.

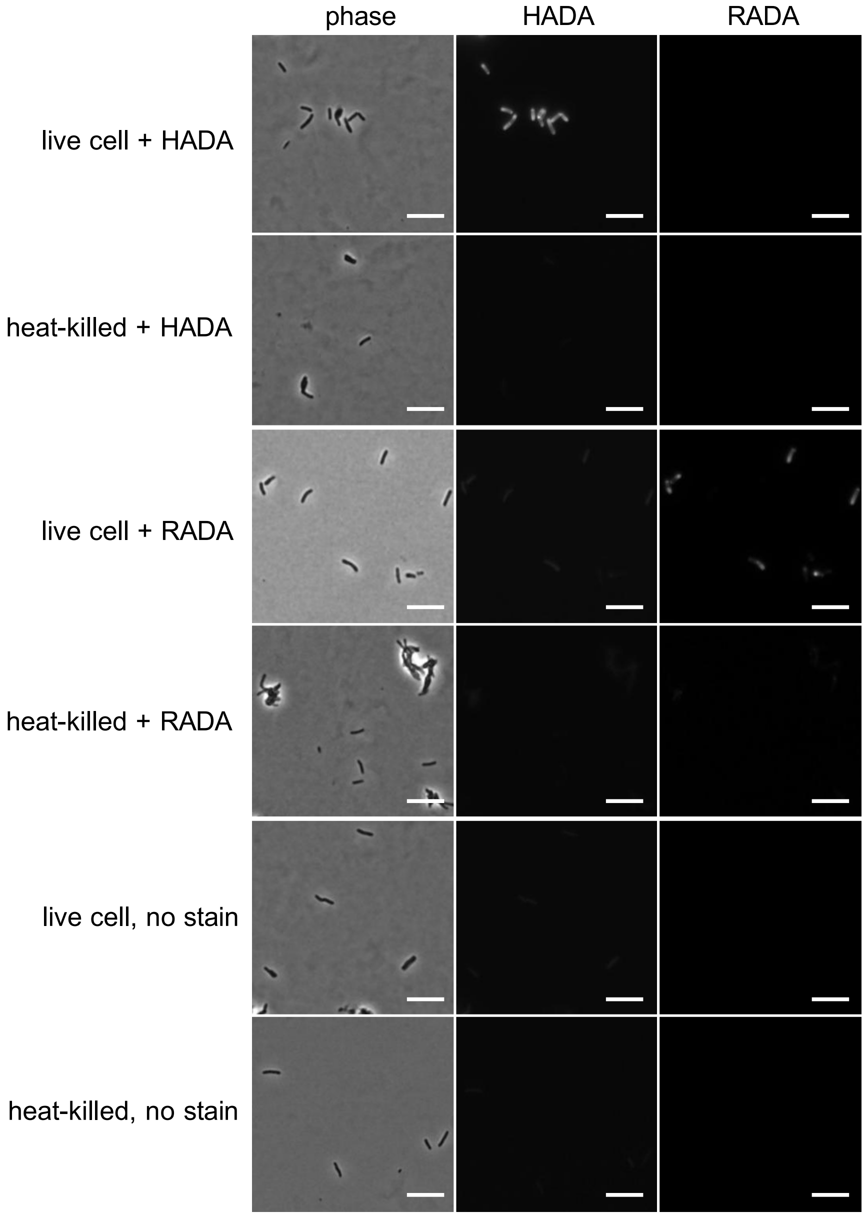
phase
HADA
RADA
live cell + HADA
heat-killed + HADA
live cell + RADA
heat-killed + RADA
live cell, no stain
heat-killed, no stain

Fig B.

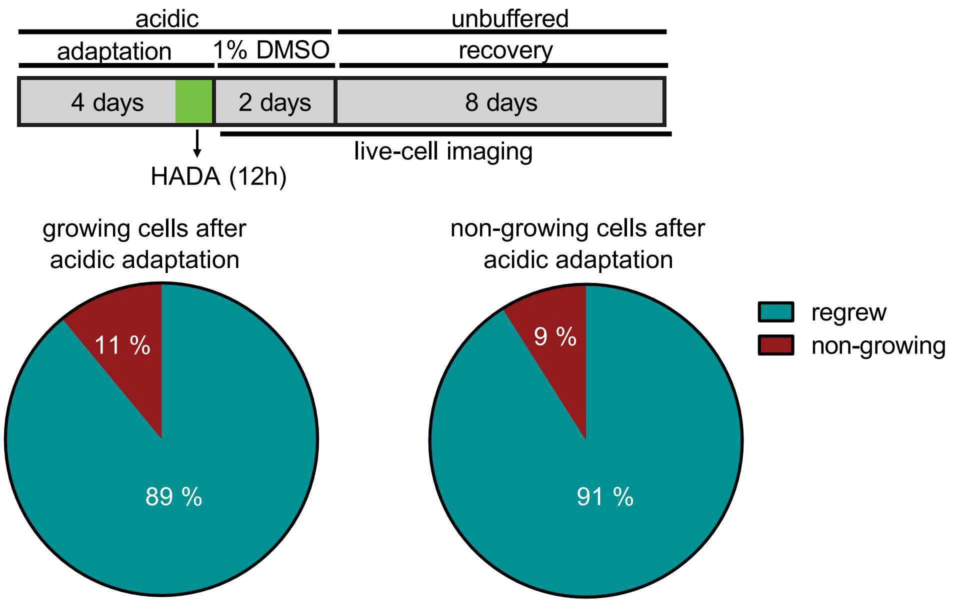

acidic
adaptation
1% DMSO
unbuffered
recovery
4 days
2 days
8 days
live-cell imaging
HADA (12h)
growing cells after
acidic adaptation
non-growing cells after
acidic adaptation
11 %
89 %
9 %
91 %
regrew
non-growing

Fig C.

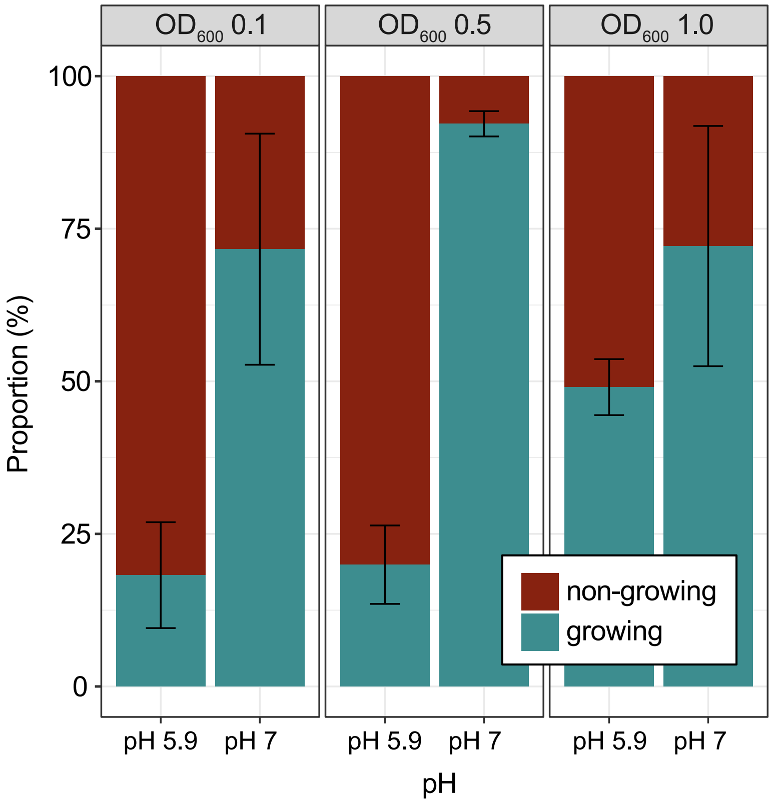

OD600 0.1
OD600 0.5
OD600 1.0
100
75
50
25
0
Proportion (%)
non-growing
growing
pH 5.9
pH 7
pH 5.9
pH 7
pH 5.9
pH 7
pH

Fig D.

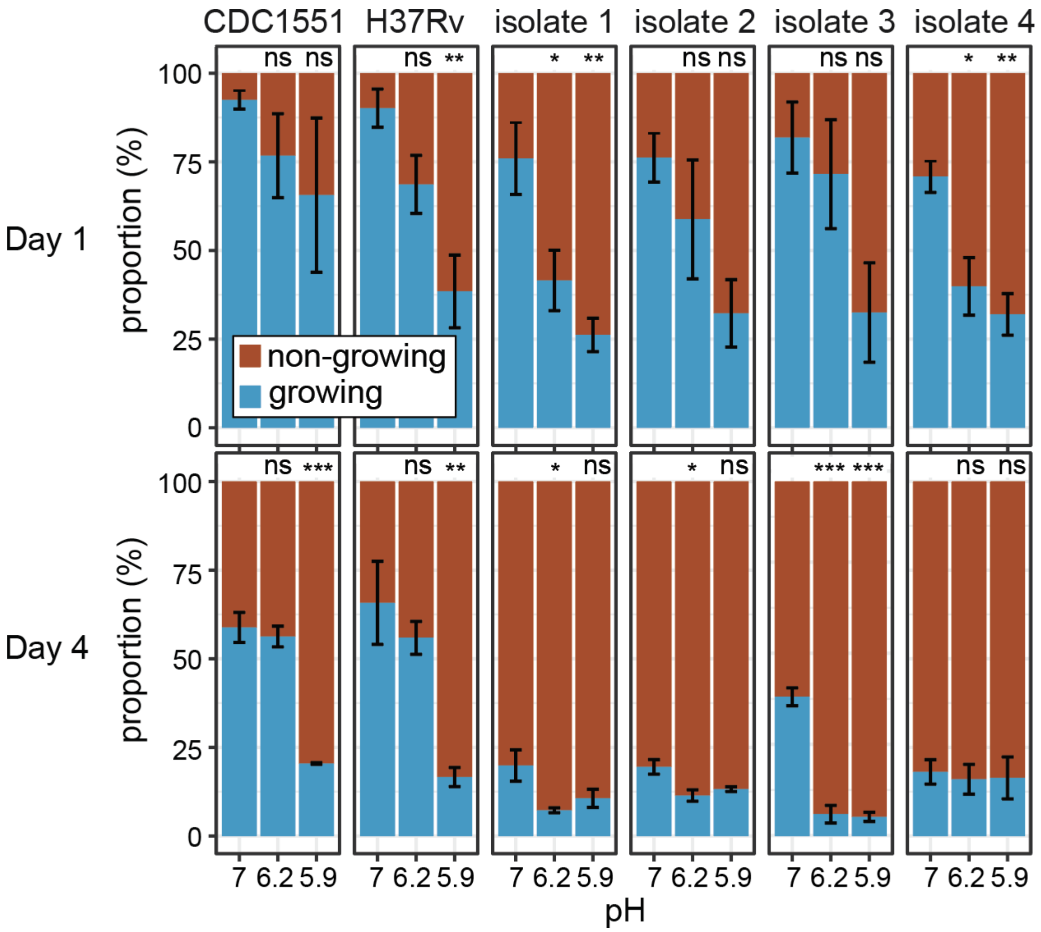
CDC1551
H37Rv
isolate 1
isolate 2
isolate 3
isolate 4
Day 1
Day 4
proportion (%)
100
75
50
25
0
ns ns
ns **
* **
ns ns
ns ns
* **
ns ***
ns **
* ns
* ns
*** ***
ns ns
non-growing
growing
7 6.2 5.9
pH

Fig E.

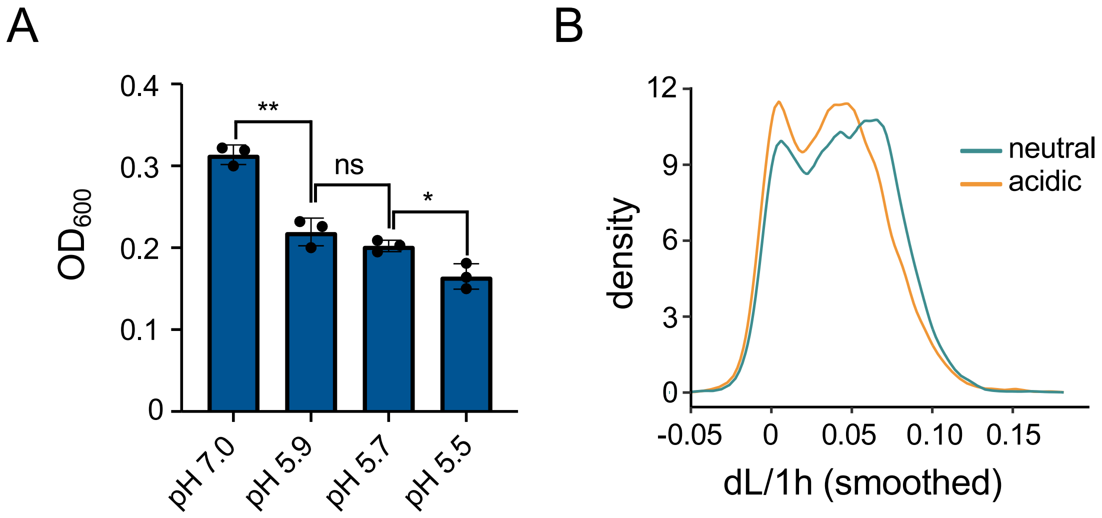
A
B
0.4
0.3
0.2
0.1
0
OD600
**
ns
*
pH 7.0
pH 5.9
pH 5.7
pH 5.5
12
9
6
3
0
density
neutral
acidic
-0.05
0
0.05
0.10
0.15
dL/1h (smoothed)

35 **Fig F.**

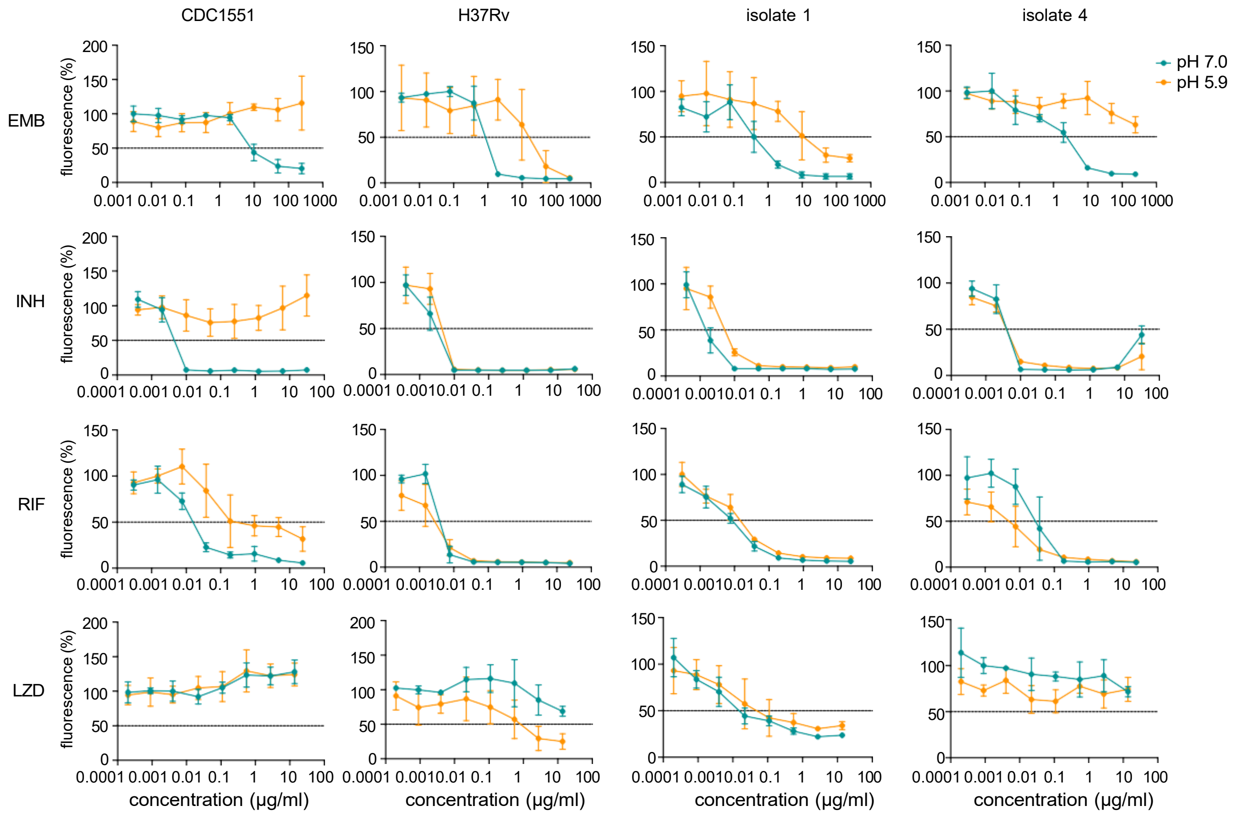

CDC1551
H37Rv
isolate 1
isolate 4
pH 7.0
pH 5.9
EMB
INH
RIF
LZD
fluorescence (%)
concentration (µg/ml)

36

Fig G.

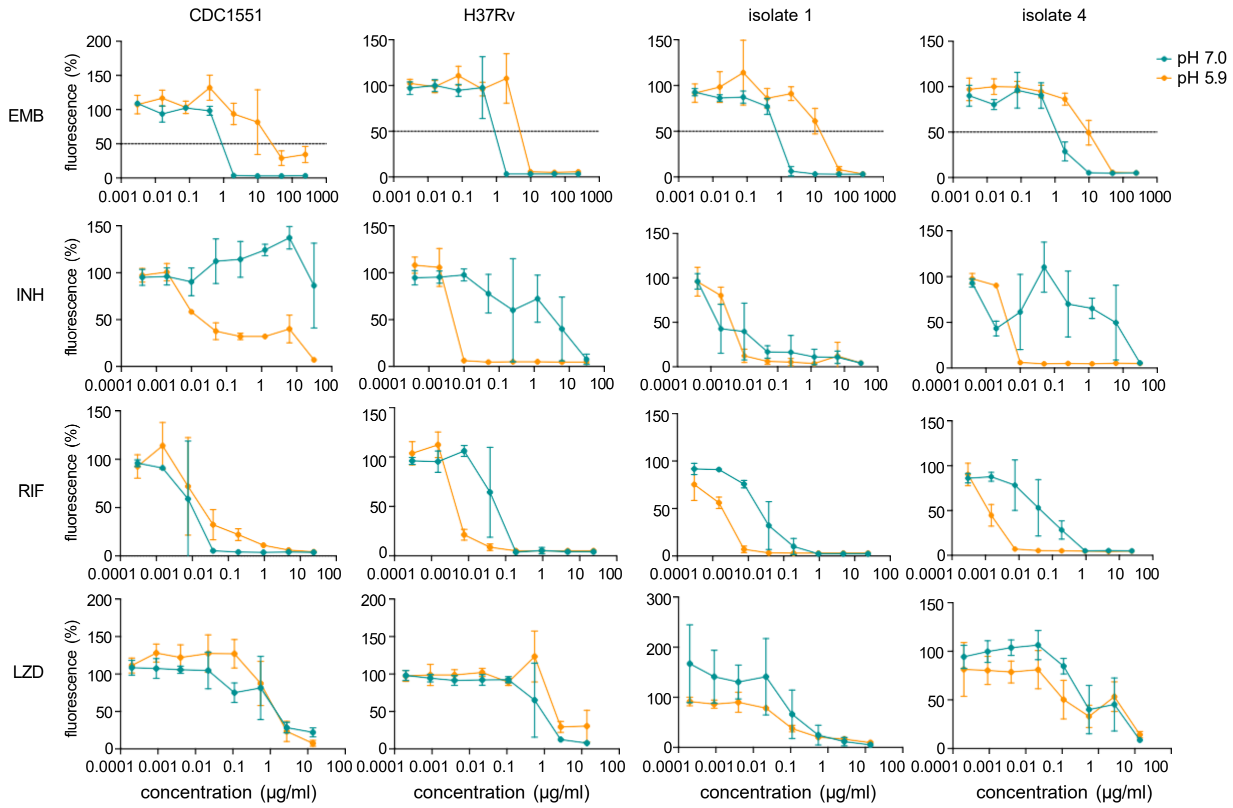
CDC1551
H37Rv
isolate 1
isolate 4
pH 7.0
pH 5.9
EMB
INH
RIF
LZD
fluorescence (%)
concentration (µg/ml)

Fig H.

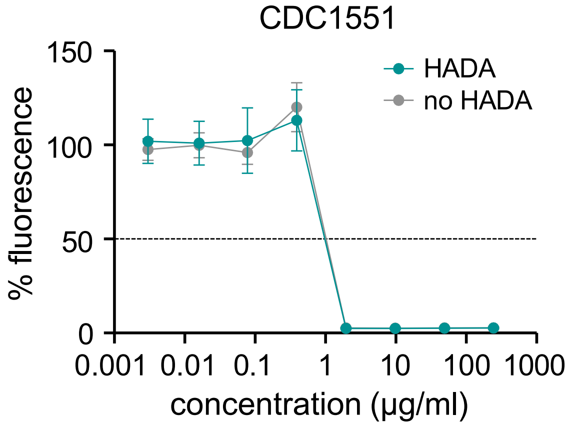
CDC1551
HADA
no HADA
% fluorescence
150
100
50
0
0.001
0.01
0.1
1
10
100
1000
concentration (µg/ml)

Fig I.

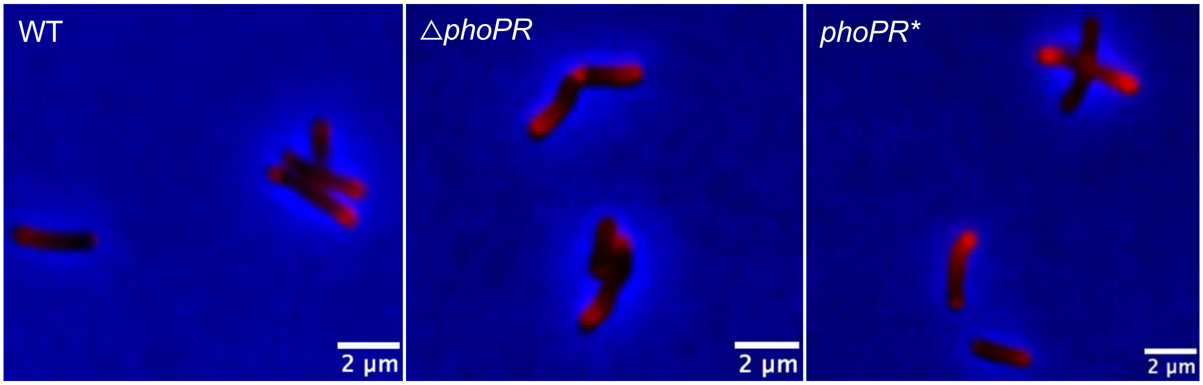

Fig J.

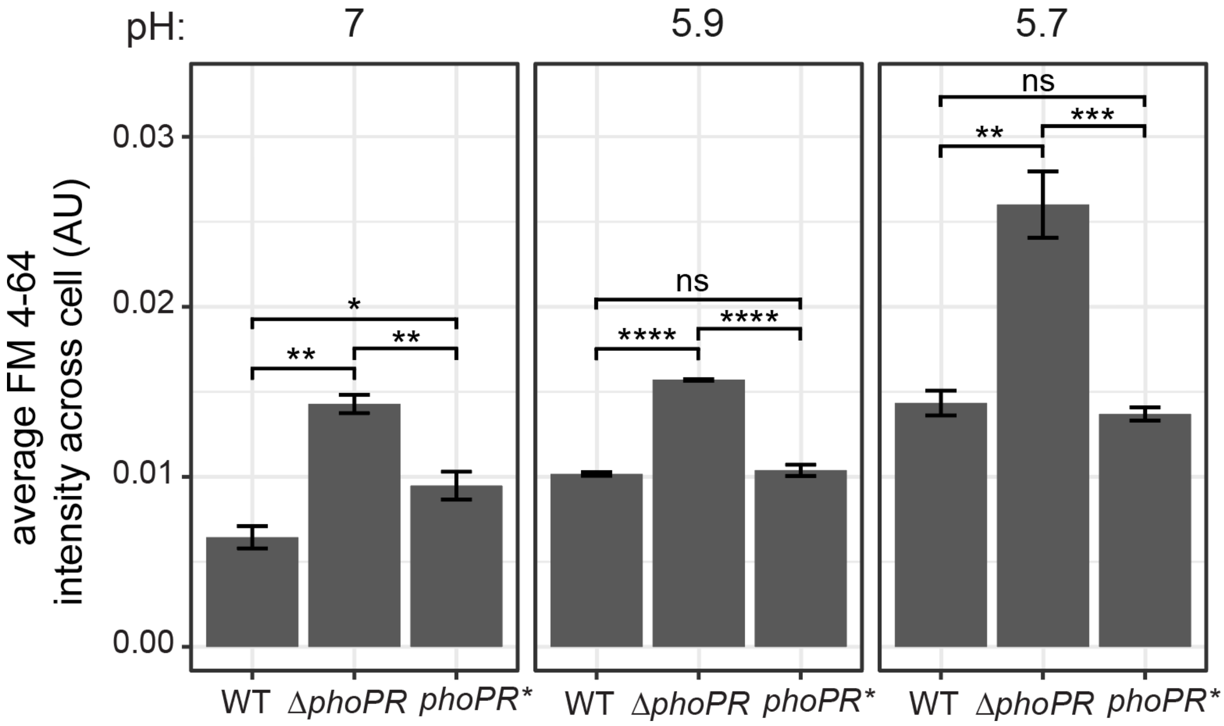

pH:
7
5.9
5.7
average FM 4-64
intensity across cell (AU)
0.00
0.01
0.02
0.03
*
**
**
ns
****
****
ns
**
***
WT
ΔphoPR
phoPR*

Table A.

	Cells recovered	Cells not recovered	Total cells counted	Percentage of cells that recovered
HADA-positive	5	731	736	0.7
HADA-negative	20	132	152	13.2
total	25	863	888	

Table B.

CDC1551 adjusted p-value	Isolate 1 adjusted p-value	Isolate 2 adjusted p-value	Gene Name	Rv#	log2 FC (fitness)
$2.8x10^{-23}$	$2.3x10^{-26}$	$3.4x10^{-06}$	Rv0412c	Rv0412c	-2.3
$5.5x10^{-69}$	$3.1x10^{-51}$	$7.1x10^{-22}$	*pknG*	Rv0410c	-2.1
$6.4x10^{-12}$	$7.4x10^{-13}$	$2.1x10^{-07}$	*embB*	Rv3795	-2
$1.1x10^{-43}$	$1.0x10^{-21}$	$4.0x10^{-23}$	Rv0996	Rv0996	-1.7
$2.6x10^{-10}$	$2.1x10^{-34}$	$3.2x10^{-11}$	*leuC*	Rv2988c	-1.3
$1.7x10^{-111}$	$5.0x10^{-111}$	$9.3x10^{-24}$	*mmpL8*	Rv3823c	-1.2
$1.3x10^{-12}$	$3.1x10^{-30}$	$3.0x10^{-12}$	*nuoN*	Rv3158	-1.2
$1.4x10^{-17}$	$7.5x10^{-127}$	$5.5x10^{-30}$	*clpB*	Rv0384c	-1.1
$6.8x10^{-11}$	$8.7x10^{-38}$	$3.1x10^{-16}$	*nuoM*	Rv3157	-1
$6.0x10^{-17}$	$1.2x10^{-16}$	$1.1x10^{-16}$	Rv1815	Rv1815	-1
